# The consequences of denoising marker-based metagenomic data

**DOI:** 10.1186/1753-6561-6-S6-P11

**Published:** 2012-10-01

**Authors:** John M Gaspar, W Kelley Thomas

**Affiliations:** 1 Department of Molecular, Cellular, and Biomedical Sciences, University of New Hampshire, Durham, NH, USA

## Background

Early marker-based metagenomic studies, such as those of the human microbiome, were performed without properly accounting for the effects of noise (pyrosequencing errors, PCR single-base errors, and PCR chimeras). One popular solution to address these issues is to utilize AmpliconNoise [1]. This collection of algorithms was validated on mock community datasets in which the 'correct' result, such as the number of operational taxonomic units (OTUs), was known. However, when conducting a real study, one will not know the correct result, but still must consider how the data has been transformed by denoising.

## Materials and methods

We applied AmpliconNoise to several real metagenomic datasets. At each stage of the pipeline, we reconstituted the reads and determined how they had been affected. The changes were quantified as substitutions, insertions, deletions and '3' gap', which is the number of bases removed from (or added to) the 3' end of a read. We further analyzed the effects of the related denoising programs in QIIME (Denoiser [2]) and in mothur [3].

## Results

The preliminary filtering steps of AmpliconNoise caused most of the sequence reads to be eliminated or truncated. Following this, the algorithm PyroNoise caused changes to the reads that were inconsistent with the known spectrum of pyrosequencing errors, until one of the parameters was increased substantially. Additionally, because PyroNoise mapped reads onto longer representatives, sequences were added to the 3' ends of reads that were often dissimilar from those that were removed by the truncations of the filtering steps. After this, SeqNoise, which was designed to remove PCR single-base errors, further clustered the reads and caused even more changes to the reads with little justification.

Denoiser, which is based on an earlier version of AmpliconNoise, caused far more changes to the data. The evaluation of the changes was not as clear here, since they were not clearly delineated as to which type of errors they were correcting, but we found some of the same flawed methodology that produced much of the negative effects seen in AmpliconNoise. This was also true of the denoising programs in mothur, which were recoded directly from the AmpliconNoise algorithms.

## Conclusions

While reducing the effects of noise in the analysis of marker-based metagenomic data is important, the algorithms of AmpliconNoise make changes to sequence reads that are inconsistent with simply removing noise. We recommend that those using AmpliconNoise be cognizant of the possible side effects and, at a minimum, consider adjusting the parameters of the algorithms accordingly.
